# Supplementary material for: SerpinB3 Upregulates Low-Density Lipoprotein Receptor-Related Protein (LRP) Family Members, Leading to Wnt Signaling Activation and Increased Cell Survival and Invasiveness
Source: Biology (Basel). 2023 May 26;12(6):771. doi: 10.3390/biology12060771 (PMC10295789; doi:10.3390/biology12060771)
Supplement: Supplementary file 1 [file biology-12-00771-s001.zip › biology-2298311-supplementary.pdf]

SUPPLEMENTARY TABLES

TABLE S1. List of the antibodies used in the study

Antibody	PROCEDURE	Dilution	Supplier
anti-LRP-1 85k Da	IF	1:50	Meridian Life Sciece, Inc, Memphis, USA
anti-LRP-1 85k Da	WB	5 μg/ml	Meridian Life Sciece, Inc, Memphis, USA
Anti-LRP-1 515 kDa	Blocking experiments	0.5-5 μg/ml	Meridian Life Sciece, Inc, Memphis, USA
Anti-LRP-5	IF	1 ug/ml	BD Bioscience, Franklin Lakes, NJ, USA
Anti-LRP-6	IF	1:1000	Cell Signaling, Danvers, MA, USA
anti-SERPINB3	IF	8 μg/ml	Hepa-Ab, Xeptagen S.p.A., Marghera, VE, Italy
anti-vimentin	IF	1:1000	GeneTex, Alton Parkway Irvine, CA, USA
anti-vimentin	WB	1:3000	GeneTex, Alton Parkway Irvine, CA, USA
anti-E-cadherin	IF	1:1000	BD Bioscience, Franklin Lakes, NJ, USA
anti-E-cadherin	WB	1:3000	BD Bioscience, Franklin Lakes, NJ, USA
Alexa Fluor® 488 Goat Anti-Mouse IgG	IF	1:500	Invitrogen Life Technologies, NY, USA
Alexa Fluor® 546 Goat Anti-Rabbit IgG	IF	1:500	Invitrogen Life Technologies, NY, USA
anti-mouse IgG Peroxidase conjugated	WB	1:1000	Sigma-Aldrich, St. Louis, MO, USA
anti-rabbit IgG Peroxidase conjugated	WB	1:1000	Sigma-Aldrich, St. Louis, MO, USA

WB: Western blot; IF: immunofluorescence

TABLE S2. List of the primers used in the study.

**Target**	**Orientation**	**Sequence (5' to 3')**
CD274	Sense	TTGCTGAACGCCCCATACAA
	Antisense	GGAATTGGTGGTGGTGGTCT
SerpinB3	Sense	GCAAATGCTCCAGAAGAAAG
	Antisense	CGAGGCAAAATGAAAAGATG
Wnt-1	Sense	CAAACAGCGGCGTCTGATAC
	Antisense	AGCCTCGGTTGACGATCTTG
Wnt-7a	Sense	AACTTGCACAACAACGAGGC
	Antisense	TTGTCCTTGAGCACGTAGCC
cMyc	Sense	AAGACAGCGGCAGCCCGAAC
	Antisense	TGGGCGAGCTGCTGTCGTTG
β-catenin	Sense	TGGTGCCCAGGGAGAACCCC
	Antisense	TGTCACCTGGAGGCAGCCCA
Axin	Sense	AACGACAGCGAGCAGCAGAG
	Antisense	AGCTTGTGACACGGCCCTGG
LRP-1	Sense	AGCAAACGAGGCCTAAGTCA
	Antisense	GCTGCTTGTGCTGATGGTAA
GAPDH	Sense	TGGTATCGTGGAAGGACTCATGAC
	Antisense	ATGCCAGTGAGCTTCCCGTTCAGC

TABLE S3. Clinical and histological characteristics of the patients included in the study**.**

**Number of patients:**	**38**
Age (mean years ± SD)	62,7 ± 9,5
Male sexex (%)	29/38 (76,3%)
**Etiology**	
HBV infection	7/38 (18,4%)
HCV infection	17/38 (44,7%)
HBV+HCV infection	3/38 (7,9%)
Alcohol use	12/38 (31,6%)
Other	5/38 (11.1%)
**Pathology**	
Number of nodules (min-max)	1,44 (1-3)
Nodule diameter mean – mm (min-max)	54,55 (7-190)
Vascular invasion	19 (50,00%)
- Microscopic	18 (47,37%)
- Macroscopic	1 (2,63%)
**Grading**	
GI	3 (7,9 %)
GII	21 (55,26%)
GIII	14 (36,84%)

SUPPLEMENTARARY FIGURES

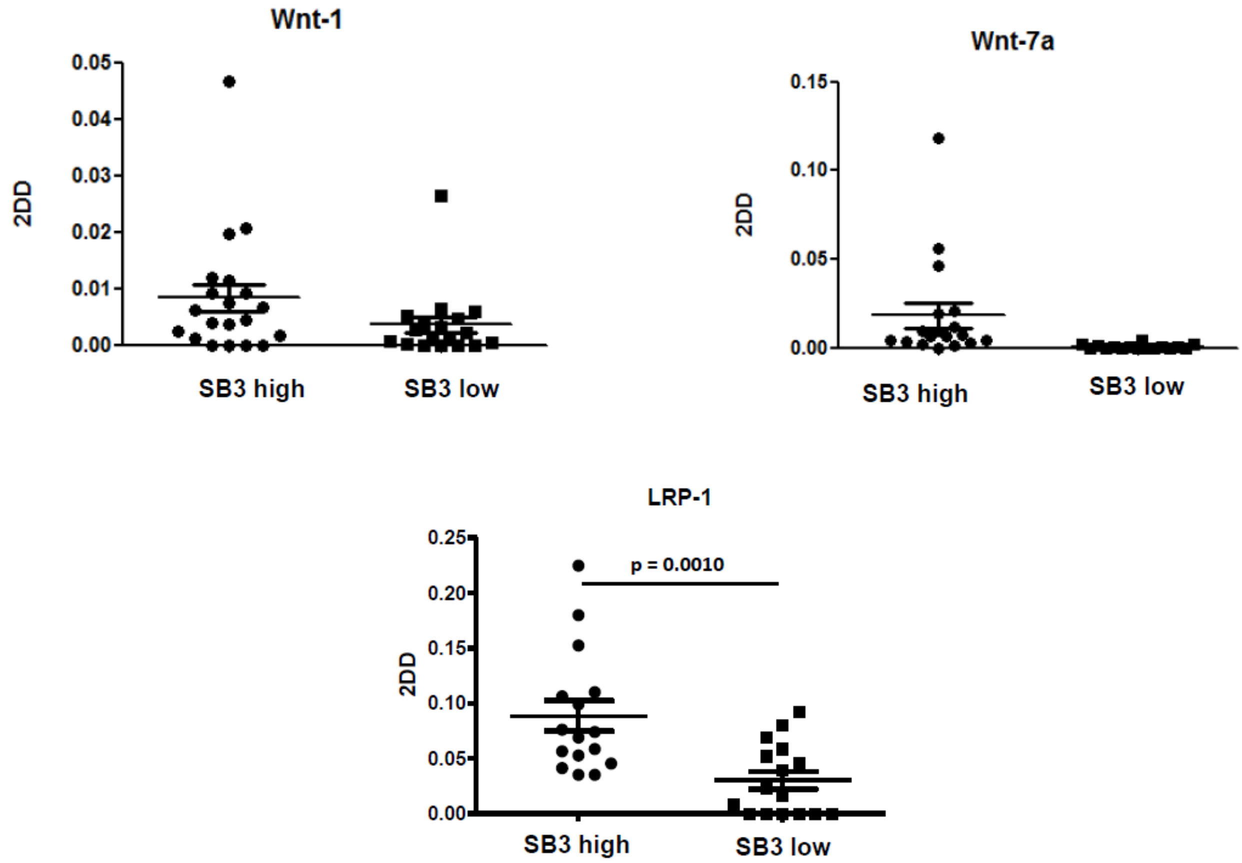

Figure S1 Relative mRNA expression of SerpinB3 compared with Wnt-1, Wnt-7a and LPR-1 in human HCCs. The expression of Wnt-1, Wnt-7a and of LPR-1 was evaluated in 38 tumor specimens from HCC patients, grouped on the basis of the expression of SB3, where high is $\geq$ median value and low is < median value. Experiments were performed in triplicate and data were expressed as mean +/- SD (vertical bars).

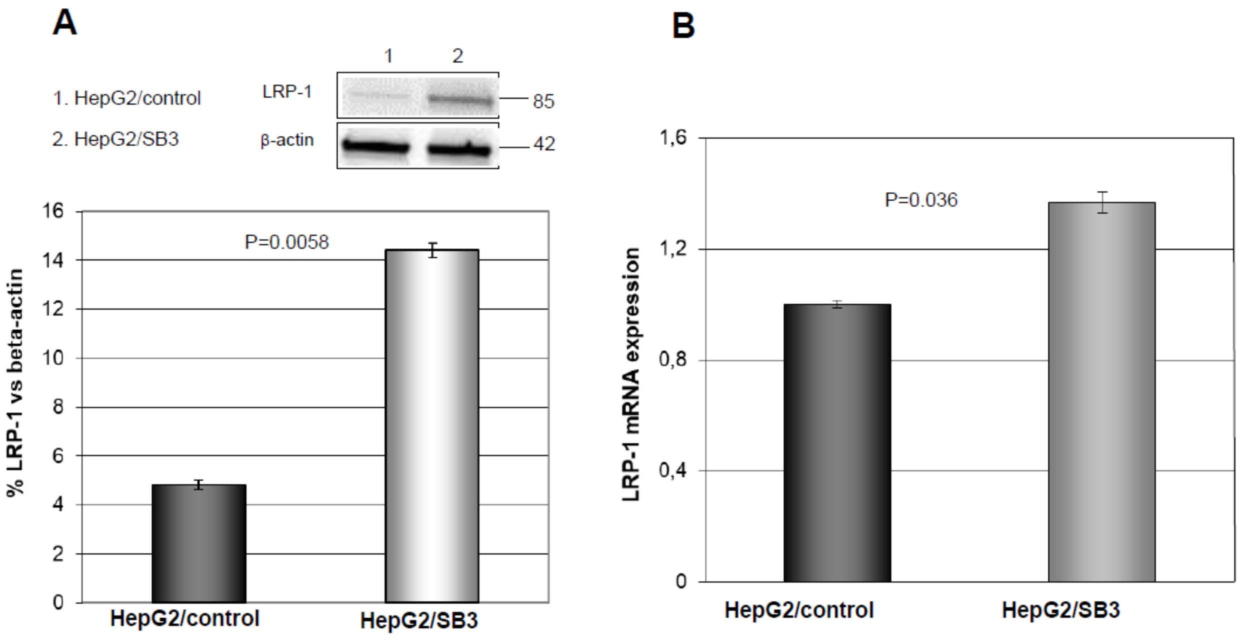

Figure S2 LRP-1 expression in hepatoma cells. A): example of densitometric analysis of Western blot results for LRP-1 protein, normalized to β-actin and the cropped blots of LRP-1 and of the housekeeping β-actin. B): relative LRP-1 mRNA expression in HepG2/SerpinB3 cells. Results are expressed as fold change (2−ΔΔCt) compared to control cells (HepG2/empty vector). Experiments were performed in triplicate and data were expressed as mean +/- SD (vertical bars).

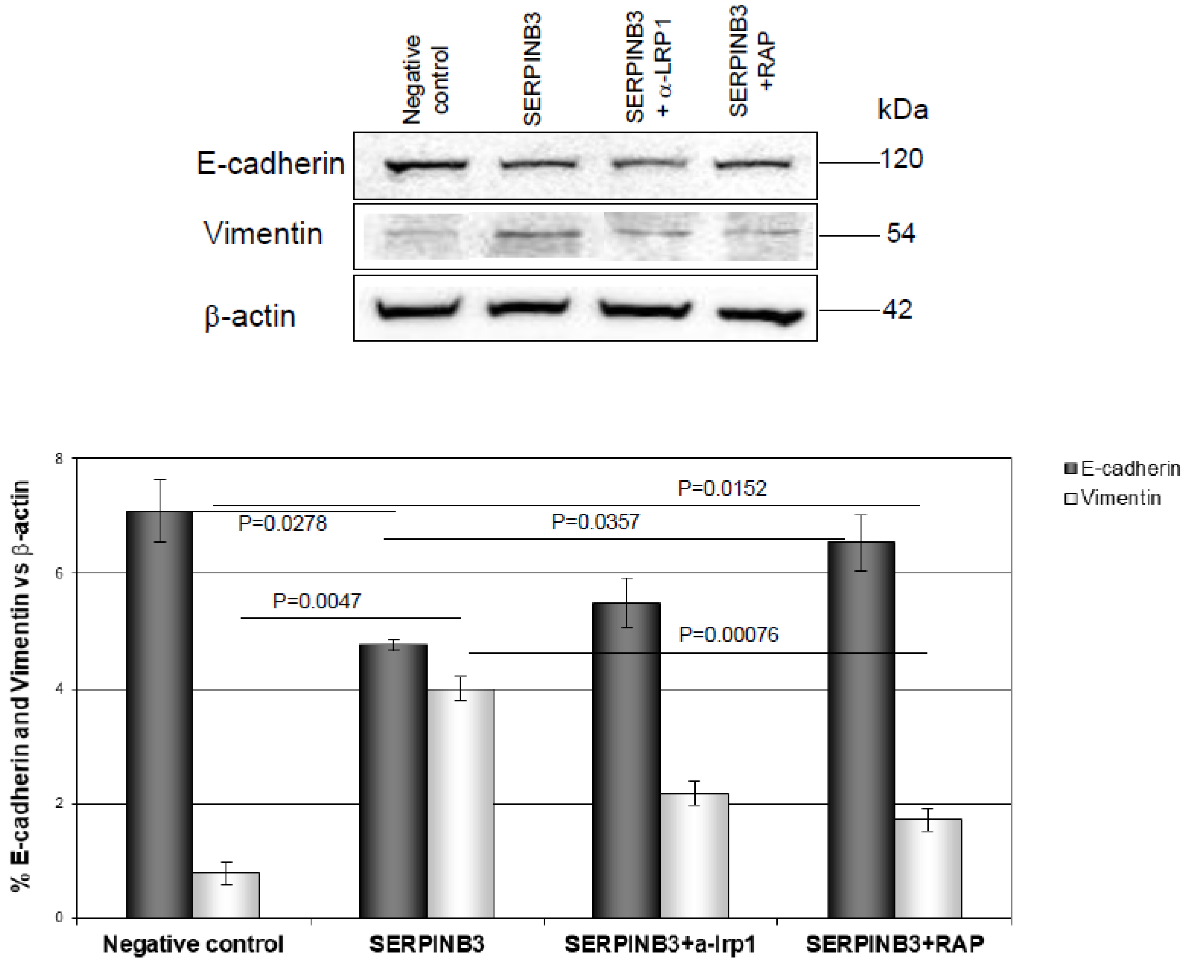

Figure S3 Western blot analysis of E-cadherin and vimentin. Upper panel: example of cropped immunoblot of E-cadherin, vimentin and of the housekeeping β-actin. Samples were loaded in the same gel and run under the same experimental conditions. Lower panel: histogram of quantitative densitometric analysis of the intensity of E-cadherin and vimentin bands, normalized to β-actin obtained in HepG2/empty vector cells incubated overnight with PBS, as negative control, with 100 ng/ml of recombinant SERPINB3 protein, as positive control. Cells were pre-treated with 5 µg/ml of anti-LRP antibody for 1 hour or overnight incubated with 5 µg/ml of RAP before treatment with 100ng/ml of recombinant SerpinB3. Experiments were performed in triplicate. Data were expressed as mean +/- SD (vertical bars).
